# EHealth to empower patients with musculoskeletal pain in rural Australia (EMPoweR) a randomised clinical trial: study protocol

**DOI:** 10.1186/s12891-020-03866-2

**Published:** 2021-01-05

**Authors:** Carlos I. Mesa-Castrillon, Milena Simic, Manuela L. Ferreira, Kristy Hatswell, Georgina Luscombe, Antonio Michell de Gregorio, Phillip R. Davis, Adrian Bauman, Stephen Bunker, Ornella Clavisi, Grahame Knox, Kim L. Bennell, Paulo H. Ferreira

**Affiliations:** 1 Musculoskeletal Health, Faculty of Medicine and Health, The University of Sydney, 75 East Street, Lidcombe, Sydney, NSW 1825 Australia; 2 Faculty of Medicine and Health, The Institute of Bone and Joint Research, The Kolling Institute, The University of Sydney, Sydney, NSW Australia; 3 Physiotherapy department, Dubbo Health Service, Western NSW Local Health District, Dubbo, NSW Australia; 4 School of Rural Health, The University of Sydney, Orange Campus, Orange, NSW Australia; 5 School of Public Health, The University of Sydney, Sydney, NSW Australia; 6 Medibank Private, Melbourne, VIC Australia; 7 Musculoskeletal Australia, Muscle Bone & Joint Health Ltd, Melbourne, Australia; 8 Physiotherapy department, Orange Health Service, Western NSW Local Health District, Orange, NSW Australia; 9 Centre for Health, Exercise and Sports Medicine, University of Melbourne, Melbourne, VIC Australia

**Keywords:** Low back pain, Knee osteoarthritis, Telehealth, eHealth, Rural health, Resistance exercise, Physical activity

## Abstract

**Background:**

Low back pain (LBP) and knee osteoarthritis (OA) are major contributors to disability worldwide. These conditions result in a significant burden at both individual and societal levels. Engagement in regular physical activity and exercise programs are known to improve physical function in both chronic LBP and knee OA populations. For people residing in rural areas, musculoskeletal conditions are often more frequent and disabling compared to urban populations, which could be the result of reduced access to appropriate health services and resources in rural settings. EHealth is an innovative solution to help provide equitable access to treatment for people with musculoskeletal pain living in rural settings.

**Methods/design:**

We will conduct a randomised clinical trial investigating the effects of an eHealth intervention compared to usual care, for people with chronic non-specific LBP or knee OA in rural Australia. We will recruit 156 participants with non-specific chronic LBP or knee OA. Following the completion of baseline questionnaires, participants will be randomly allocated to either the eHealth intervention group, involving a tailored physical activity and progressive resistance exercise program remotely delivered by a physiotherapist (*n* = 78), or usual care (n = 78) involving referral to a range of care practices in the community. Outcomes will be measured at baseline, 3 and 6 months post-randomisation. The primary outcome will be physical function assessed by the Patient-Specific Functional Scale (PSFS). Secondary outcomes include pain intensity, physical activity levels, activity limitations, quality of life, pain coping. We will also collect process evaluation data such as recruitment rate, attendance and adherence, follow-up rate, participants’ opinions and any barriers encountered throughout the trial.

**Discussion:**

The findings from this trial will establish the effectiveness of eHealth-delivered interventions that are known to be beneficial for people with LBP and knee OA when delivered in person. As a result, this trial will help to inform health care policy and clinical practice in Australia and beyond for those living in non-urban areas.

**Trial registration:**

This study was prospectively registered on the Australian New Zealand Clinical Trials Registry (ACTRN12618001494224) registered 09.05.2018.

## Background

Low back pain (LBP) and knee osteoarthritis (OA) are among the highest contributors to disability worldwide when diseases are ranked according to the number of Years Lived with Disability (YLD) [1, 2]. The outlook is often worse for people living in rural areas where musculoskeletal conditions can be more prevalent and disabling compared to urban populations [3–6]. In 2011 rural Australians were 23% more likely to report LBP, when compared to those living in major cities [7], and the prevalence of OA in any joint is higher in regional centres (25%) and remote areas (23%) compared with major cities (19%) [8]. Rural populations have reduced access to health services and facilities to manage these conditions [9–11], and therefore wait in pain longer to receive treatment with a median waiting time of 120 days in rural, compared to 103 in urban regions (interquartile range, 55–204 and 42–210, respectively) [10]. While the reasons for this disparity are multidimensional, including socioeconomic and cultural factors, the distance required to travel for health care is a common barrier to appropriate and timely healthcare in rural areas [12].

Clinical guidelines commonly recommend the implementation of regular physical activity and exercise programs to improve physical function in people with chronic LBP [13] and knee OA [14]. Previous research has also shown that engagement in progressive resistance strengthening exercises is both safe and beneficial for patients with chronic LBP and knee OA [15–17]. Allied health professionals (such as physiotherapists) commonly design and deliver this type of intervention during face-to-face consultations. However, people residing in non-urban areas often experience health workforce shortages, especially the availability of musculoskeletal specialists which substantially decline with increasing rurality [12, 18]. This scenario can delay appropriate and timely evidence-based first-line management and prevention of symptom deterioration in rural populations. Therefore, innovative models of delivery of health care services are needed, to secure equitable access to recommended treatment for chronic musculoskeletal conditions irrespective of where patients reside.

EHealth is defined as ‘health services and information delivered or enhanced through the internet and related technologies’ [19]. It is widely recognised for its potential to minimise travel costs and inconvenience for patients when accessing health services in rural areas [20–23]. There is growing evidence that eHealth is feasible and effective in remote delivery of health services to patients with long-term conditions including chronic obstructive pulmonary disease [24], spinal cord injury [25], multiple sclerosis [26], and chronic pain [27].

The recommended conservative management for both chronic LBP and knee OA, which commonly coexist, include: 1) physical activity engagement, and 2) exercise therapy [13, 14]. However, the effectiveness of a comprehensive patient-centred physical activity and exercise intervention delivered through eHealth, and specially designed for patients with chronic LBP or knee OA who live in rural communities has not been previously investigated.

## Methods

### Aim

The primary aim of this study is to investigate the effectiveness of a three-month physiotherapist-delivered eHealth intervention comprised of a tailored physical activity plan and progressive resistance exercise program, to improve physical function in people living in rural Australia with chronic non-specific LBP or knee OA, compared to usual care.

The secondary aim is to evaluate the effectiveness of the intervention to improve pain intensity, activity limitations, quality of life, physical activity levels, and pain coping compared to usual care. We will also collect process evaluation data such as recruitment rate, attendance and adherence, follow-up rate, participants’ opinions and any difficulties or barriers encountered throughout the trial. We hypothesise that the eHealth intervention will be effective and will result in better levels of physical function compared to usual care.

### Study design

This study will be a Phase II randomised clinical trial, including a parallel design. This protocol has been designed according to the Standard Protocol Items: Recommendations for Interventional Trials (SPIRIT) statement [28]. For a completed SPIRIT checklist, see the online Additional file 1.

### Participants and setting

We will recruit 156 participants nation-wide from rural regions across Australia and the outpatient physiotherapy department at Dubbo Hospital in the Western New South Wales Local Health District of Australia. To be included in the trial, potential participants must present with one of the following:

- Non-specific LBP lasting for at least three months; have experienced back pain intensity ≥3/10 on the 11-point numeric pain rating scale in the past month, and be aged ≥18 years old.
- Knee OA, with symptoms lasting for at least three months; have knee pain ≥3/10 on the 11-point numeric pain rating scale over most days of the past month, and be aged ≥45 years.

Participants who report both conditions will be stratified based on their primary complaint by reporting which condition concerns them the most. Non-specific LBP and knee OA will be diagnosed after screening red flags and excluding specific conditions using recommended criteria from the clinical practice guidelines provided by the American College of Physicians and the American Pain Society [29], and the National Institute for Health and Clinical Excellence (NICE) guidelines [30], respectively.

To be included, participants must also meet all of the following criteria:

- Seeking (or planning to in the next three months) care which incur costs to patients and/or health care system, for their back or knee pain;
- Reside in rural Australia areas based on the Modified Monash Model (MM) classification 2019 (MM 2 to MM 7) [31];
- Fluent in English (verbal and written);
- Adequate hearing and eyesight;
- Independent ambulatory status;
- Current internet access;
- Own an internet-capable device with a display, camera, microphone, and speaker (smartphone, tablet or computer);
- Access the internet at least once a month;
- Have a self-rated ability to use the internet as at-least fair (using a 4-item scale from poor, fair, good to excellent) [32];

Potential participants will be excluded if they have any of the following:

- Any recent or imminent spinal or knee surgery (within 12 months), or had knee replacement surgery for their OA;
- Any corticosteroid injections on the knee or spinal joints in the past month;
- Evidence of radiculopathy, nerve root, spinal cord, or cauda equine compression.
- LBP caused by involvement in a road traffic accident in the last 12 months or ongoing litigation;
- Presence of a comorbid condition that would prevent active participation in performing strengthening exercises at home or increasing physical activity levels;
- Diagnosis of fibromyalgia or a systematic arthritic condition;
- Current or recent pregnancy;
- Recent fall history deemed to impose a risk for potential injury;

### Participant timeline

**Fig. 1:**
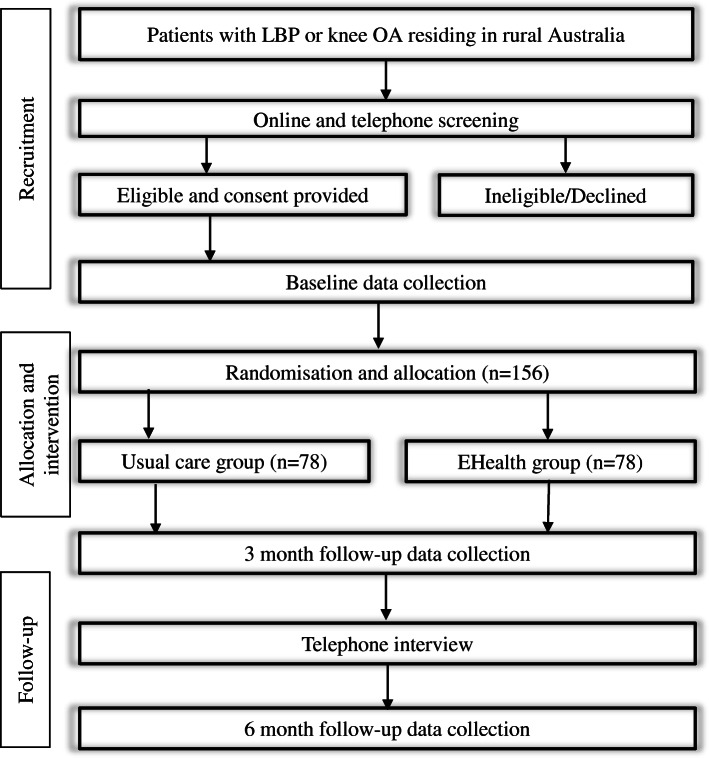
Flowchart of the Empower study

Table 1 shows the assessments at each time point following the Standard Protocol Items: Recommendations for Interventional Trials (SPIRIT) statement [28]. Figure 1 demonstrates the flow chart of the study.

**Table 1:**
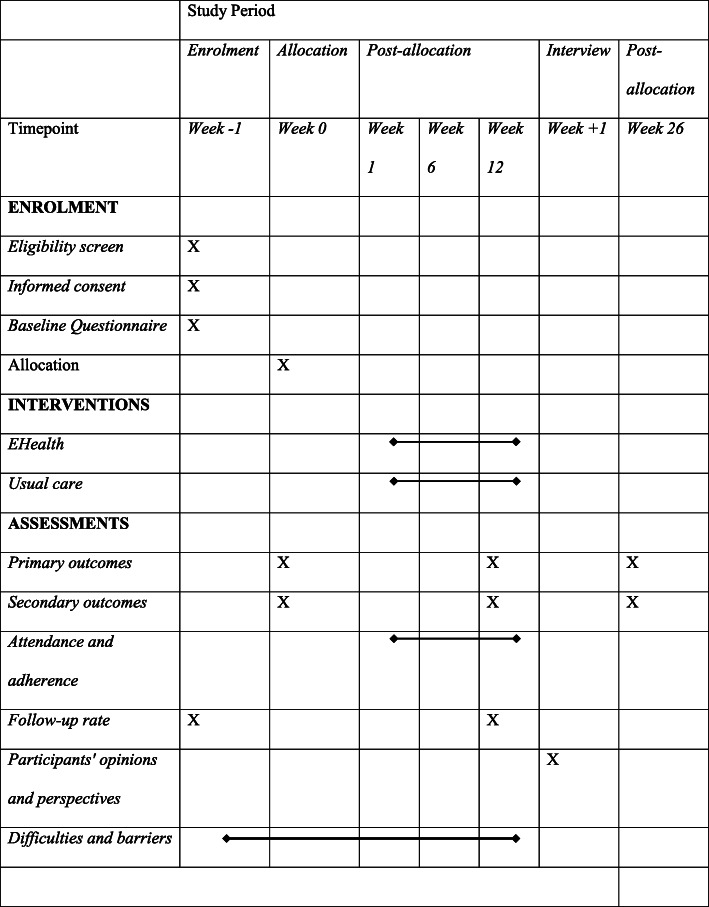
Study assessments at specific time points.

### Recruitment procedure

Participants will be recruited from the general community across Australia and the outpatient department at the Dubbo Hospital, Western New South Wales (NSW) Health District, NSW, Australia. All the trial procedures (e.g. eligibility screening, randomisation, baseline and follow-up questionnaires and study interventions) will be performed online.

Participants living in rural communities in Australia who are intending to seek care for their symptoms will be sought through the use of social media and printed advertisements. Advertisement flyers will be distributed through the University of Sydney platforms and relevant external organisations across Australia such as rural organisations, networks, councils, e-newsletter, etc. The online method of delivery will include email newsletters and social media platforms, including Facebook, and Instagram. Hard copies of advertisement flyers will be posted on public notice boards, placed in local businesses, and handed out at events (e.g. NSW Country Women’s Association) in rural Australia where potential participants can be identified. Study advertisements will direct individuals to a study website or to contact the research team to receive further information.

Individuals who are seeking care will also be identified from an outpatient physiotherapy department at a regional hospital in Dubbo, NSW. Individuals will be identified from the hospital’s waiting list or recent intake forms and contacted by hospital staff during a routine telephone call to schedule an appointment to be provided with information about the trial. If interested and verbal consent is provided, individual contact details will be entered into a secure REDCap (Research Electronic Data Capture) web survey by hospital staff. Individuals will then be directed to an online screening questionnaire, followed by a telephone screening to confirm eligibility, participant information sheet, consent form, and online baseline questionnaire.

### Randomisation and allocation

Randomisation will be blinded and performed using a computer-generated random allocation schedule operated centrally at the University of Sydney by a remote research assistant using the REDCap web application. Participants, hospital staff, and the eHealth physiotherapist will be notified, via automatic REDCap emails, about which group the participant has been randomly allocated. For participants recruited from the Dubbo hospital, a total timeframe of one week will be required to complete the recruitment and randomisation process.

### Blinding

Participants and physiotherapists will not be blinded to group allocation and will be aware of the alternative treatment components, but study hypotheses will not be disclosed to participants. The chief trial coordinator, the research assistant conducting baseline and follow-up collection, and statistician will be blinded to treatment allocation.

### EHealth intervention

Participants allocated to the eHealth intervention will schedule their first remote video consultation by email or telephone with the eHealth physiotherapist. The intervention includes a physical activity plan and a progressive resistance exercise program designed during remote video consultations with a physiotherapist using the Physitrack® Ltd., LDN, UK (physiotherapist end), and Physiapp® (participant end) web applications. Participants will be equipped with (i) a welcome booklet with an overview of the intervention components; (ii) instructions on how to use the Physiapp® web application (Web browser, Android or iOS app) at home; and (iii) an exercise kit consisting of a range of resistance bands. The intervention will be delivered over three months and guided by a maximum total of eight remotely-delivered video consultations in real-time with a physiotherapist. The initial consultation will last up to one hour, while subsequent consultations will be 30 min. The physiotherapist and patient will agree on the number of consultations according to the patient’s availability, with a minimum of 5 and a maximum of 8 video consultations being planned [33].

#### The home exercise program

During the physiotherapy video consultations, a home-exercise program will be designed according to patients’ preferences, individual goals, and the performance of remotely supervised exercises. The initial exercise prescription will have a dosage of two sets of 8–15 repetitions; 2 to 3-min rest in between sets; up to 4 total exercises to promote early adherence [34–36]; with a frequency of 3 times a week. Selected exercises will target the knee extensors and trunk musculature in both groups before further individualisation is made. After two weeks of familiarisation [37, 38] to promote early adherence, exercises will be performed to ≥5/10 (hard) on the modified Borg Rating of Perceived Exertion scale [39]. Resistance will be provided by using body weight or resistance bands to modify the difficulty of an exercise. The exercise dose used in this study is based on both the American College of Sports Medicine’s minimum recommendations for maintaining health [40] and previous studies using a similar intensity and frequency in non-specific chronic LBP and knee OA populations [17, 37, 39, 41, 42]. Modified exercises and dose will be provided for participants that have difficulties reaching this dosage initially or if, for example, muscular endurance is more appropriate. A 5–10 min warm-up and cool-down period will be included before and after each home exercise session focusing on joint range of motion and relaxation, respectively. Participants will be able to see the details of the exercise program, access video resources, record the details of each session and view their progress using the Physiapp® web application. Subsequent video consultations will allow to the physiotherapist to modify or make a progression of exercises if appropriate. Treatment fidelity will be assessed using a treatment note checklist within 24 h after each remote physiotherapy consultation. Table 2 shows a summary of how the exercise program is designed. Further details of the home-exercise program procedure can be found in online Additional file 2.

**Table 2:** Home Exercise Program

**Exercise selection**	
Key prescription factors	• Patient goals • Patient preferences • Activity limitations
Body region	• At-least 1 exercise targeting the knee extensors • At-least 1 exercise targeting the trunk
Amount of exercises	• Minimum of 2 and Maximum of 4 exercises (initially)
Equipment used	• Patient surroundings, i.e. furniture, outdoor environment, access to any other exercise equipment such as a bike • Theraband® elastic band colour green
**Exercise dosage**	
Approximate intensity	• According to patient tolerance and symptomatology to exercise
Rated Perceived Exertion (RPE)	• At-least 5/10 RPE (hard)
Repetitions	• 8–15
Sets	• 2–3
Rest between sets	• 2–3 min
Frequency	• Three days per week (non-consecutive days)
Time period	• 3 months
Total consultation	• ≥5 consultations out of 8 offered
Familiarisation period	• Initial two weeks
**Modifications**	
Alternative	• Alternative dosage (if appropriate) - Isometric (30–60 s total hold time) - Endurance (15–25 repetitions)
Progression	• When the patient consistently over-performs above the prescribed dosage • Only one change at a time made when progressing
Simplification	• When the patient consistently underperforms below prescribed dosage (at least three consecutive sessions)
Examples of progression or simplification	• Adding or removing an exercise • Replacing an existing exercise • Changing the level of resistance band • Increasing or reducing isometric pauses • Modify surroundings or body position

#### The physical activity plan

The physical activity plan will be implemented according to the principles used in our previous study of physical activity for musculoskeletal pain [43]. The focus of the program will be on a gradual increase in physical activity where participants will be encouraged to devise fortnightly goals to suit and advance their physical activity levels. Each physiotherapist will be trained in the principles of health coaching to facilitate the process. The plan will be specifically tailored to each participant and will involve goal setting, monitoring, and progression of goals using the web application. Physical activity is prescribed according to the health coaching SMARTS goals, and it is tailored to each participant preference on the type of physical activity and functionality. It is also based on the World Health Organization guidelines, and they are not a goal itself but a desirable objective that will vary according to the participant’s previous physical activity levels and pain intensity. Further details of the physical activity plan can be found in online Additional file 2.

### Usual care group

The usual care provided in this trial is described as de facto usual care and includes the wide range of care practices provided in a community which are unrestricted by study protocols or rules [44]. This is in contrast to usual standardised care which may not reflect real-life care provided in the participating rural communities [44]. Due to the nature of de facto usual care and the dual recruitment strategy (general and hospital patient communities) used in this trial, therapy received by participants in this group will be self-reported using a weekly logbook throughout the study period. The specific treatments and care providers involved will vary depending on the participant’s location and source of recruitment. The common practice is likely to involve a visit to a primary care professional (e.g. GP, physiotherapist), prescription of pain medication, education, home exercises, and if within close vicinity to a health site, some additional supervised exercises tailored to the individual and may or may not involve additional manual therapy techniques.

All participants allocated into the usual care group will be contacted via telephone and email by a member from the research team who will liaise with the participant about what the planned care they were seeking for their LBP or knee OA. Participants recruited from the Dubbo Hospital outpatient physiotherapy department will have an appointment already scheduled and therefore, will only require a reminder and instructions to continue as usual. Participants recruited from the general community will be asked a series of questions about the health care site they were intending to access before entering the study. Assistance will be provided to those who are unaware of what options are available in their local community via a health service directory supported by the governments of Australia (https://www.healthdirect.gov.au/). A referral letter will then be provided to each participant, and the respective health care site mutually decided beforehand. Costs may be incurred to participants depending on their care pathway.

### Outcome measures

Outcome measures will be taken through an online link via REDCap at baseline, at 3 and 6 months follow-up post-randomisation.

#### Primary outcome

1. Physical function assessed using the Patient-Specific Functional Scale (PSFS) [45]; which ask participants to list three activities that they are unable to do are having difficulties because of their musculoskeletal pain. Each listed activity is then rated on an 11—point difficulty scale from 0 (‘unable to perform activity’) to 10 (‘100% able to perform the activity’). A total score ranging from 0 and 30 is calculated with higher scores indicating higher levels of physical function. This tool is valid and reliable for the evaluation of physical function in both people with chronic LBP [45] and those with knee OA [46].

#### Secondary outcomes

2. LBP or knee pain intensity over the past week assessed using an 11-point numeric rating scale (NRS) [47];
3. Condition-specific activity limitation assessed using the Roland-Morris Disability Questionnaire (RDQ) [48] for LBP or the Western Ontario and McMaster Osteoarthritis Index (WOMAC) [49] for knee OA;
4. Health-related quality of life measured using the Assessment of Quality of Life (AQoL)-8D instrument [50];
5. Physical activity level assessed using the short-form of the International Physical Activity Questionnaire – Short Form (IPAQ-SF) [51];
6. Pain self-efficacy measured using the Pain self-efficacy questionnaire [52].

#### Process evaluation measures

1. Measures of recruitment rate: during the recruitment stage of the trial, records will be kept regarding the number of participants screened for entry to the trial. If patients do not meet the study’s inclusion criteria, the reason they were ineligible for inclusion will be recorded. Similarly, if eligible, the reasons for declining participation in the trial will be noted.
2. Measures of adherence: Both groups will receive a study paper-based logbook to record treatment details, medication use, adverse events, and health care travel details (See online Additional file 3). Participants will be provided with reply-paid envelopes for return of logbooks or will be provided with the opportunity to send them electronically. Participants in the eHealth intervention will be prescribed a total of 36 home exercise sessions over three months (three sessions per week). We will consider a self-reported number of completed exercise sessions converted to a percentage of total prescribed sessions. We will consider completion of 70% of prescribed home exercise sessions during the three months to be acceptable adherence [39].
3. Measures of attendance: completion of the video consultation will be monitored and recorded by the eHealth physiotherapist. Participants in the control group will also report their attendance to a health care professional through the paper-based logbook.
4. The measure of follow-up rate: the number of participants in each group who complete the baseline and follow-up assessments will be recorded. The reasons for the drop-out of participants will be recorded when possible throughout the different phases of this trial.
5. The measure of opinion and perspectives: a phone interview will be used to investigate participants’ opinions and perspectives in both the eHealth and usual care group regarding the study. All participants who completed the study and those who dropped out will be invited to participate in the audio-recorded interview. They will be asked to rate their satisfaction on a scale from 0 (‘lowest satisfaction level’) to 10 (‘highest level of satisfaction’) regarding:
  i) Overall experience with the study;
  ii) Accessibility of reaching the health professional
  iii) Time to get an appointment with the health professional
  iv) Cost of the intervention received
  v) The distance needed to travel for health care
  vi) Only participants in the eHealth group will be asked to rate their satisfaction with the Physiapp platform and exercise equipment provided.
  vii) Finally, all participants will be asked whether they would recommend the care to someone else.
6. Difficulties and barriers to completing the trial: any issue experienced throughout the study will be recorded.

#### Other outcomes

7. The research team will closely monitor self-reported adverse events defined as increased musculoskeletal pain, trip/fall, serious events or other musculoskeletal symptoms assessed using a standardised weekly logbook. It will be recorded if the adverse event lasted more than 24 h and whether the participant required medical attention. Participants will be instructed to contact the study project manager if they believe any adverse event was caused by participating in the study.
8. Medication and other health care use will be assessed using a weekly logbook.
9. Distance travelled in kilometres to utilise health care (summed for all types of health care visits related to musculoskeletal pain) during the three month intervention period will be collected using the weekly logbook.

### Data integrity and monitoring

An independent Data Safety Monitoring Committee (DSC) (an independent researcher not a member in the coauthor’s group) will be assembled and responsible for assessing trial safety and ensuring that the best interests of participants are observed at all times. A single data quality audit will be performed during the study period, once 50% of the sample size is reached.

Furthermore, data will be stored in password-protected spreadsheets on secured servers hosted by the University of Sydney, which will be regularly scrutinised for omissions and errors, and then transferred to appropriate statistical software for analysis by an investigator blinded to group allocation. All the data collected will be restricted to the lead investigator and selected members of the research team using the backend of the project. A REDCap code will be created to allow re-identification when necessary.

### Sample size

Sample size calculation was based on the between-group difference of 3 points on our primary outcome, i.e. PSFS (0–30 points)) which has been reported as the scale’s minimum clinically important difference (MCID) for both Knee OA and LBP [53, 54]. In our previous study of physical activity and exercises for LBP, the response within each subject group on physical function was normally distributed with a standard deviation of 7 [54]. A sample size of 78 per group (total of 156 participants) will achieve 80% power (Type I error of 0.05) to detect an MCID of 3 points on physical function [55] (adjusted for scale score range of 0–30), allowing for a loss to follow-up rate of 15% at six months. Calculations were performed with Stata IC/13.0 power two means function.

### Statistical analysis

Descriptive characteristics, as well as baseline outcome measures, will be used to assess baseline comparability between groups. Analyses will be by intention to treat and performed by a blinded statistician. No attempt will be made to impute missing values. The focus of the analyses will be on effect estimation rather than formal hypothesis testing. The following covariates will be accounted in the models if found to be different between groups: the effect of baseline pain and disability levels, symptom length, comorbidities, and age. The effect of group allocation on continuous outcomes (e.g. function, physical activity) will be assessed through group*time interaction terms using linear mixed models with baseline value as covariate (mixed command in Stata 15). A subgroup analysis stratified by condition (LBP or OA) will be performed separately. A statistical analysis plan will be made publicly available before analysis.

### Protocol amendments

Any modifications to the protocol will be submitted to the Human Research Ethics Committee before implementation and amendments will be communicated to the trial registries and outlined at the study dissemination.

### Confidentiality

The confidentiality of participants and the privacy of data will be protected during all publications and presentations. Data will be presented as summary statistics such that individual participants will not be identifiable in the research reports or presentations.

### Ancillary and post-trial care

Despite the efforts of the research team to mitigate risks associated with the intervention, potential small harms may occur. If participants are harmed by taking part in this research project, there are no special compensation arrangements; however, they are encouraged to contact the Human Research Ethics Committee.

### Dissemination policy

Participants will be provided with a short report summarising their results collected throughout the study. The study results will be reported on manuscripts that will be submitted to peer-reviewed journals and through the partners’ organisations involved in the trial (e.g. Medibank, The Country Women’s Association, Musculoskeletal Australia, NSW senior’s card) to be available to the community as publications. All investigators will be considered as authors of future publications according to their contribution. The protocol of the study will be available to the public on the website of the clinical trials registrations and Ethics Research Committee.

### Patient and public involvement

Patients and their community are central to dissemination about the study, which helped to motivate community involvement during and beyond the recruitment. Musculoskeletal Australia, the main patient group organisation in Australia, was involved in the study design and has supported the participation of the community in the research. Once the trial has been published, participants will be informed of the results through our website (https://www.theempowerstudy.com/) and will be sent details of the results via email in a non-technical language.

## Discussion

We present a protocol for a randomised clinical trial involving a 3-month eHealth intervention for participants with non-specific chronic LBP or knee OA living in rural Australia compared to usual care. Innovative models of care delivery are needed to address the reduced access to health services and increased health burden in many rural communities, both locally and globally. LBP and knee OA represents the top leading causes of disability worldwide costing billions of dollars to economies every year. In Australia, chronic back pain accounted, in 2008–9, for nearly 1.2 billion in healthcare expenditure [56] and a similar economic impact is evident for chronic OA, costing an estimated 3.5 billion in 2015–16 [8].

If proven to be effective, this new model of care has the potential to optimise management of a range of other chronic conditions such as heart disease and diabetes in rural settings that benefit from improved physical activity, progressive resistance training, and self-management strategies.

Results from the trial will inform rural and primary health care policy and clinical practice in Australia and beyond by establishing the effects of a novel mode of delivery of interventions that are known to be beneficial when delivered in-person to people with non-specific chronic LBP and knee OA. A potential limitation of the eHealth intervention deployed in this study is the technological skill, internet access available in rural Australia and attitudes towards eHealth, which could act as a barrier to some individuals. However, the future improvements currently underway to increase internet access to underserved areas and growing acceptance of technology in this patient population is encouraging.

## Supplementary Information

**Additional file 1.**

**Additional file 2.**

**Additional file 3.**

## Data Availability

Not applicable.

## Abbreviations

LBP: Low back pain
OA: Osteoarthritis
YLD: Years of life living with a disability
SPIRIT: Standard Protocol Items: Recommendations for Interventional Trials
NSW: New South Wales
NICE: National Institute for Health and Clinical Excellence
REDCap: Research Electronic Data Capture
PSFS: Patient-Specific Functional Scale
NRS: Numeric rating scale
RDQ: Roland-Morris Disability Questionnaire
WOMAC: Western Ontario and McMaster Osteoarthritis Index
AQoL: Assessment of Quality of Life
IPAQ-SF: International Physical Activity Questionnaire – Short Form
MCID: minimum clinically important difference
