# Supplementary material for: EHealth to empower patients with musculoskeletal pain in rural Australia (EMPoweR) a randomised clinical trial: study protocol
Source: BMC Musculoskelet Disord. 2021 Jan 5;22:11. doi: 10.1186/s12891-020-03866-2 (PMC7783996; doi:10.1186/s12891-020-03866-2)
Supplement: Supplementary file 2 — Additional file 2. [file 12891_2020_3866_MOESM2_ESM.docx]

**Additional file 3.
Exercise prescription dosage guidelines**

The focus of the home exercise program will be on progressive resistance training (PRT) using the American College of Sport Medicine’s minimum recommendations for maintaining musculoskeletal health. The exercises chosen will be individually tailored to each participant with the goal of reaching the following dosage parameters by the end of the intervention period:

- Exercises targeting the trunk and lower limb musculature (at-least one exercise targeting the knee extensors for participants with knee osteoarthritis)
- Isotonic contractions (concentric and eccentric)
- Intensity: • According to patient tolerance and symptomatology to exercise
- 2-3 sets (cycle of repetitions)
- 2-3 minutes rest between sets
- 2-3 sessions per week
- Resistance provided by a) bodyweight b) resistance bands c) cuff weights or d) other weights if available
- 5-10 minute warm up covering joint range of motion (dynamic stretching) and relaxed deep breathing

The following additional modifications might be made depending on a) individual irritability and b) individual goals if deemed appropriate by the treating physiotherapist and participant:

- Isometric contractions using 2-6 sets to complete 30-60 seconds of total contraction time
- Endurance repetition range between 15-25 repetitions

The initial home-exercise program will meet the following additional criteria to promote early adherence and minimise discomfort:

- No more than 4 exercises in total
- Exercise repetitions performed to ≈80% (2-3 reps less) of the average repetitions performed during the initial supervised consultation for a period of 2 weeks (familiarisation period). For example an exercise performed to 10 repetitions will be reduced to 8 repetitions.

**2. Steps in choosing exercises and dosage during initial consultation**

1. 5 minute warm up performed
2. Physiotherapist will discuss participant goals, preferences and past experience to give an indication for appropriate exercises (overlaps with physical activity goal setting).
3. Physiotherapist will choose an exercise video to stream for the participant to watch in real-time
4. Physiotherapist will ask the participant to perform the exercise until it becomes hard (≥5/10) using the modified Borg Rating of Perceived Exertion scale (verbally).

- Participants will be reminded they can stop the exercise when they like with verbal consent obtained prior to attempting the exercise.
- Reassurance of safety, encouragement to focus on the exercise (opposed to pain) will be provided by the physiotherapist.

1. Physiotherapist will provide feedback on performance (i.e. body position, technique, environment setup) as appropriate
2. Depending on steps (4-8) the physiotherapist may:
3. Select the exercise if the participant performs between 8-15 repetitions in two sets (2 minutes rest between)
4. Applies modification or provides new exercise if the participant performs outside 8-15 repetitions
5. Repeat steps (2-8) until a) up to 4 appropriate exercises are selected b) time runs out (allowing 5 minutes to answer questions and schedule next consultation)
6. Physiotherapist will then use the selected exercises to create the home exercise program using the Physitrack ® application ensuring:
7. Exercise sessions are spread out on non-consecutive days
8. Meets the exercise prescription guidelines in section 1
9. Warm-up and cool-down included

**Steps in modifying exercises and/or choosing new exercises during a subsequent consultation**

1. Participant self-reported adherence will be monitored using the Physitrack® application.

- If adherence is considered high/adequate for 2 weeks the progression in step 2 will be made
- If adherence is considered low the program will repeat or regression provided until adherence is met. Barriers (i.e. difficulty, concerns, symptoms, or time management) preventing adherence will be discussed).

1. Repetitions will be progressed to the average performed during the initial consultation.
2. Exercise progression from this point forward will meet the following criteria:

- Only one of the following parameters modified at a time; number of repetitions, sets, exercises or intensity (use of body weight, resistance band or cuff weight).
- Participant performs more than 12-15 repetitions consistently (2 consecutive sessions) or level of exertion drops below <5/10. This will be monitored through Physitrack® and participant feedback.
- New exercises to follow the same steps in section 2

1. Exercise regression will be made if the following criteria is met:

- Participant performs below 8 reps consistently (2 consecutive sessions) or barriers (i.e. difficulty, concerns, symptoms, or time management) prevent adherence. This be will monitored through Physitrack® and participant feedback.

**Physical activity selection guidelines.**

The physical activity program will focus on gradually increasing physical activity levels where participants will be encouraged to devise long term (up to 2) and short-term goals (fortnightly) to suit and advance their lifestyle. The physiotherapist will keep in mind the a) World Health Organisation (WHO) guidelines recommending at-least 150minutes of moderate intensity or 75 minutes of vigorous intensity aerobic physical activity or equivalent combination per week and b) the nature and normal clinical course of the participants’ condition(s). Each goal will be specific (self-set), measurable, agreed upon, realistic and time based (SMART). Goals may involve sport, leisure, work, transport, a home-exercise program or other incidental activities to reduce sedentary behaviour.

**Step in creating and progressing Physical activity program**

1. The physiotherapist (trained in health coaching) will discuss and reach mutually agreed upon SMART short-term and long-term physical activity goals (1-2 each max) with the participant. Problem-solving and suggestions will be provided if the participant finds this difficult.
2. The physiotherapist will record each goal on a PDF template before uploading to their program for participants to view.
3. The short-term goals will be included in their Physiapp® program along with the home-exercises where adherence can be recorded.
4. If the specific physical activity is not found on the Physitrack® library, custom physical activities can be created.
5. Progression or modification of goals will be made in subsequent consultations as necessary (short term goals achieved or barriers preventing achieving goals explored).
