# Supplementary material for: EHealth to empower patients with musculoskeletal pain in rural Australia (EMPoweR) a randomised clinical trial: study protocol
Source: BMC Musculoskelet Disord. 2021 Jan 5;22:11. doi: 10.1186/s12891-020-03866-2 (PMC7783996; doi:10.1186/s12891-020-03866-2)
Supplement: Supplementary file 3 — Additional file 3. [file 12891_2020_3866_MOESM3_ESM.docx]

**Empower pilot study Logbook**

- Your participation in our study is very valuable, please fill-up the following information constantly and send us pictures or copies of it fortnightly through the telephone or email below (see instructions on page 15).
- We have added an extra envelope and a postage stamp, so you can use it to send us the logbook back after 12 weeks.
- If you have any questions, please don’t hesitate to call or email: Antonio Michell, 0434934198, [empower.study@sydney.edu.au](mailto:empower.study@sydney.edu.au)
- Below you can find a list of codes you can use to complete section 2 (blue) and 3 (red) of the logbook:

**Activity codes:**

WALK= walking and treadmill

JOG= jogging

EX=calisthenics, aerobics, weights

LG= lawn, garden

DAN= dance

STR= stretching exercises

CC= child care

YOG= yoga

HH=household

SPORT= (specify)

OTHER= (specify)

**Clinician code:**

| GP | Orthopaedist | Physio | Massagist | Chiropractic | Psychologist | Other |
| --- | --- | --- | --- | --- | --- | --- |
| **GP** | **ORTHO** | **PHYS** | **MASSAG** | **CHIRO** | **PSYCH** | **(Specify)** |

|  | Week 1 _(example)_  **Date:** |
| --- | --- |
| Healthcare | 1. **Write in each column, in the corresponding day of the week, the clinician code (see page 1) you visited on this week and the kilometres travelled**  \|  \| Monday \| Tuesday \| Wednesday \| Thursday \| Friday \| Saturday \| Sunday \| \| --- \| --- \| --- \| --- \| --- \| --- \| --- \| --- \| \| **Clinician code** \|  \|  \|  \|  \|  \|  \|  \| \| **Kilometres travelled** \|  \|  \|  \|  \|  \|  \|  \|   For clinician code please see **page 1** |
|  |  |
| Exercises | 1. **Did you perform any exercises prescribed by a clinician on this week? (Tick all days that apply)**  \| Monday \| Tuesday \| Wednesday \| Thursday \| Friday \| Saturday \| Sunday \| \| --- \| --- \| --- \| --- \| --- \| --- \| --- \| \|  \|  \|  \|  \|  \|  \|  \| |
|  |  |
| Physical activity | 1. **Write in each column, in the corresponding day of the week, the physical activity code (see page 1) you performed for more than 10 minutes and the time in minutes for each activity on this week:**  \|  \| Monday \| Tuesday \| Wednesday \| Thursday \| Friday \| Saturday \| Sunday \| \| --- \| --- \| --- \| --- \| --- \| --- \| --- \| --- \| \| **Activity Code** \|  \|  \|  \|  \|  \|  \|  \| \| **Minutes** \|  \|  \|  \|  \|  \|  \|  \|   For activity codes please see **page 1** |
| F |  |
| Medication | 1. **Write in each column a medication name, strength per unit (e.g. 500 mg) please specify milligrams (mg) or grams (g) and the number of pills taken on this week:**  \|  \| Monday \| Tuesday \| Wednesday \| Thursday \| Friday \| Saturday \| Sunday \| \| --- \| --- \| --- \| --- \| --- \| --- \| --- \| --- \| \| **Medication name** \|  \|  \|  \|  \|  \|  \|  \| \| **Strength p.u.** \|  \|  \|  \|  \|  \|  \|  \| \| **N. of pills per day** \|  \|  \|  \|  \|  \|  \|  \| |
|  |  |
| Adverse events | Please add a tick (✔) to as many as appropriate:  **Did you experience any of the following this week?**  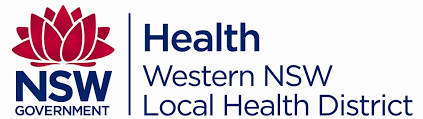Increased back pain ☐. Increased knee pain ☐. Pain elsewhere ☐. Muscle soreness ☐. Swelling ☐. Cramp ☐.  Trip/fall ☐. Serious event ☐. Other symptoms ☐  **Did any of the above last more than 24 hours?** Yes ☐**.** No ☐.  **Did you require medical attention?** Yes ☐**.** No ☐. If you feel that participation in this study caused any of the above or are concerned, please contact the project manager on 0434934198. If it is an emergency please call 000. |
|  | Week 1  **Date:** |
| Healthcare | 1. **Write in each column, in the corresponding day of the week, the clinician code (see page 1) you visited on this week and the kilometres travelled**  \|  \| Monday \| Tuesday \| Wednesday \| Thursday \| Friday \| Saturday \| Sunday \| \| --- \| --- \| --- \| --- \| --- \| --- \| --- \| --- \| \| **Clinician code** \|  \|  \|  \|  \|  \|  \|  \| \| **Kilometres travelled** \|  \|  \|  \|  \|  \|  \|  \|   For clinician code please see **page 1** |
|  |  |
| Exercises | 1. **Did you perform any exercises prescribed by a clinician on this week? (Tick all days that apply)**  \| Monday \| Tuesday \| Wednesday \| Thursday \| Friday \| Saturday \| Sunday \| \| --- \| --- \| --- \| --- \| --- \| --- \| --- \| \|  \|  \|  \|  \|  \|  \|  \| |
|  |  |
| Physical activity | 1. **Write in each column, in the corresponding day of the week, the physical activity code (see page 1) you performed for more than 10 minutes and the time in minutes for each activity on this week:**  \|  \| Monday \| Tuesday \| Wednesday \| Thursday \| Friday \| Saturday \| Sunday \| \| --- \| --- \| --- \| --- \| --- \| --- \| --- \| --- \| \| **Activity Code** \|  \|  \|  \|  \|  \|  \|  \| \| **Minutes** \|  \|  \|  \|  \|  \|  \|  \|   For activity codes please see **page 1** |
| F |  |
| Medication | 1. **Write in each column a medication name, strength per unit (e.g. 500 mg) please specify milligrams (mg) or grams (g) and the number of pills taken on this week:**  \|  \| Monday \| Tuesday \| Wednesday \| Thursday \| Friday \| Saturday \| Sunday \| \| --- \| --- \| --- \| --- \| --- \| --- \| --- \| --- \| \| **Medication name** \|  \|  \|  \|  \|  \|  \|  \| \| **Strength p.u.** \|  \|  \|  \|  \|  \|  \|  \| \| **N. of pills per day** \|  \|  \|  \|  \|  \|  \|  \| |
|  |  |
| Adverse events | Please add a tick (✔) to as many as appropriate:  **Did you experience any of the following this week?**  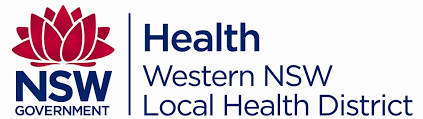Increased back pain ☐. Increased knee pain ☐. Pain elsewhere ☐. Muscle soreness ☐. Swelling ☐. Cramp ☐.  Trip/fall ☐. Serious event ☐. Other symptoms ☐  **Did any of the above last more than 24 hours?** Yes ☐**.** No ☐.  **Did you require medical attention?** Yes ☐**.** No ☐. If you feel that participation in this study caused any of the above or are concerned, please contact the project manager on 0434934198. If it is an emergency please call 000. |
|  | Week 2  **Date:** |
| Healthcare | 1. **Write in each column, in the corresponding day of the week, the clinician code (see page 1) you visited on this week and the kilometres travelled**  \|  \| Monday \| Tuesday \| Wednesday \| Thursday \| Friday \| Saturday \| Sunday \| \| --- \| --- \| --- \| --- \| --- \| --- \| --- \| --- \| \| **Clinician code** \|  \|  \|  \|  \|  \|  \|  \| \| **Kilometres travelled** \|  \|  \|  \|  \|  \|  \|  \|   For clinician code please see **page 1** |
|  |  |
| Exercises | 1. **Did you perform any exercises prescribed by a clinician on this week? (Tick all days that apply)**  \| Monday \| Tuesday \| Wednesday \| Thursday \| Friday \| Saturday \| Sunday \| \| --- \| --- \| --- \| --- \| --- \| --- \| --- \| \|  \|  \|  \|  \|  \|  \|  \| |
|  |  |
| Physical activity | 1. **Write in each column, in the corresponding day of the week, the physical activity code (see page 1) you performed for more than 10 minutes and the time in minutes for each activity on this week:**  \|  \| Monday \| Tuesday \| Wednesday \| Thursday \| Friday \| Saturday \| Sunday \| \| --- \| --- \| --- \| --- \| --- \| --- \| --- \| --- \| \| **Activity Code** \|  \|  \|  \|  \|  \|  \|  \| \| **Minutes** \|  \|  \|  \|  \|  \|  \|  \|   For activity codes please see **page 1** |
| F |  |
| Medication | 1. **Write in each column a medication name, strength per unit (e.g. 500 mg) please specify milligrams (mg) or grams (g) and the number of pills taken on this week:**  \|  \| Monday \| Tuesday \| Wednesday \| Thursday \| Friday \| Saturday \| Sunday \| \| --- \| --- \| --- \| --- \| --- \| --- \| --- \| --- \| \| **Medication name** \|  \|  \|  \|  \|  \|  \|  \| \| **Strength p.u.** \|  \|  \|  \|  \|  \|  \|  \| \| **N. of pills per day** \|  \|  \|  \|  \|  \|  \|  \| |
|  |  |
| Adverse events | Please add a tick (✔) to as many as appropriate:  **Did you experience any of the following this week?**  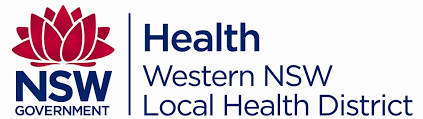Increased back pain ☐. Increased knee pain ☐. Pain elsewhere ☐. Muscle soreness ☐. Swelling ☐. Cramp ☐.  Trip/fall ☐. Serious event ☐. Other symptoms ☐  **Did any of the above last more than 24 hours?** Yes ☐**.** No ☐.  **Did you require medical attention?** Yes ☐**.** No ☐. If you feel that participation in this study caused any of the above or are concerned, please contact the project manager on 0434934198. If it is an emergency please call 000. |
|  | Week 3  **Date:** |
| Healthcare | 1. **Write in each column, in the corresponding day of the week, the clinician code (see page 1) you visited on this week and the kilometres travelled**  \|  \| Monday \| Tuesday \| Wednesday \| Thursday \| Friday \| Saturday \| Sunday \| \| --- \| --- \| --- \| --- \| --- \| --- \| --- \| --- \| \| **Clinician code** \|  \|  \|  \|  \|  \|  \|  \| \| **Kilometres travelled** \|  \|  \|  \|  \|  \|  \|  \|   For clinician code please see **page 1** |
|  |  |
| Exercises | 1. **Did you perform any exercises prescribed by a clinician on this week? (Tick all days that apply)**  \| Monday \| Tuesday \| Wednesday \| Thursday \| Friday \| Saturday \| Sunday \| \| --- \| --- \| --- \| --- \| --- \| --- \| --- \| \|  \|  \|  \|  \|  \|  \|  \| |
|  |  |
| Physical activity | 1. **Write in each column, in the corresponding day of the week, the physical activity code (see page 1) you performed for more than 10 minutes and the time in minutes for each activity on this week:**  \|  \| Monday \| Tuesday \| Wednesday \| Thursday \| Friday \| Saturday \| Sunday \| \| --- \| --- \| --- \| --- \| --- \| --- \| --- \| --- \| \| **Activity Code** \|  \|  \|  \|  \|  \|  \|  \| \| **Minutes** \|  \|  \|  \|  \|  \|  \|  \|   For activity codes please see **page 1** |
| F |  |
| Medication | 1. **Write in each column a medication name, strength per unit (e.g. 500 mg) please specify milligrams (mg) or grams (g) and the number of pills taken on this week:**  \|  \| Monday \| Tuesday \| Wednesday \| Thursday \| Friday \| Saturday \| Sunday \| \| --- \| --- \| --- \| --- \| --- \| --- \| --- \| --- \| \| **Medication name** \|  \|  \|  \|  \|  \|  \|  \| \| **Strength p.u.** \|  \|  \|  \|  \|  \|  \|  \| \| **N. of pills per day** \|  \|  \|  \|  \|  \|  \|  \| |
|  |  |
| Adverse events | Please add a tick (✔) to as many as appropriate:  **Did you experience any of the following this week?**  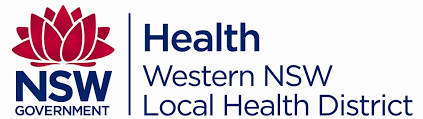Increased back pain ☐. Increased knee pain ☐. Pain elsewhere ☐. Muscle soreness ☐. Swelling ☐. Cramp ☐.  Trip/fall ☐. Serious event ☐. Other symptoms ☐  **Did any of the above last more than 24 hours?** Yes ☐**.** No ☐.  **Did you require medical attention?** Yes ☐**.** No ☐. If you feel that participation in this study caused any of the above or are concerned, please contact the project manager on 0434934198. If it is an emergency please call 000. |
|  | Week 4  **Date:** |
| Healthcare | 1. **Write in each column, in the corresponding day of the week, the clinician code (see page 1) you visited on this week and the kilometres travelled**  \|  \| Monday \| Tuesday \| Wednesday \| Thursday \| Friday \| Saturday \| Sunday \| \| --- \| --- \| --- \| --- \| --- \| --- \| --- \| --- \| \| **Clinician code** \|  \|  \|  \|  \|  \|  \|  \| \| **Kilometres travelled** \|  \|  \|  \|  \|  \|  \|  \|   For clinician code please see **page 1** |
|  |  |
| Exercises | 1. **Did you perform any exercises prescribed by a clinician on this week? (Tick all days that apply)**  \| Monday \| Tuesday \| Wednesday \| Thursday \| Friday \| Saturday \| Sunday \| \| --- \| --- \| --- \| --- \| --- \| --- \| --- \| \|  \|  \|  \|  \|  \|  \|  \| |
|  |  |
| Physical activity | 1. **Write in each column, in the corresponding day of the week, the physical activity code (see page 1) you performed for more than 10 minutes and the time in minutes for each activity on this week:**  \|  \| Monday \| Tuesday \| Wednesday \| Thursday \| Friday \| Saturday \| Sunday \| \| --- \| --- \| --- \| --- \| --- \| --- \| --- \| --- \| \| **Activity Code** \|  \|  \|  \|  \|  \|  \|  \| \| **Minutes** \|  \|  \|  \|  \|  \|  \|  \|   For activity codes please see **page 1** |
| F |  |
| Medication | 1. **Write in each column a medication name, strength per unit (e.g. 500 mg) please specify milligrams (mg) or grams (g) and the number of pills taken on this week:**  \|  \| Monday \| Tuesday \| Wednesday \| Thursday \| Friday \| Saturday \| Sunday \| \| --- \| --- \| --- \| --- \| --- \| --- \| --- \| --- \| \| **Medication name** \|  \|  \|  \|  \|  \|  \|  \| \| **Strength p.u.** \|  \|  \|  \|  \|  \|  \|  \| \| **N. of pills per day** \|  \|  \|  \|  \|  \|  \|  \| |
|  |  |
| Adverse events | Please add a tick (✔) to as many as appropriate:  **Did you experience any of the following this week?**  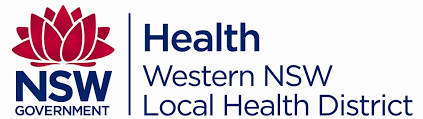Increased back pain ☐. Increased knee pain ☐. Pain elsewhere ☐. Muscle soreness ☐. Swelling ☐. Cramp ☐.  Trip/fall ☐. Serious event ☐. Other symptoms ☐  **Did any of the above last more than 24 hours?** Yes ☐**.** No ☐.  **Did you require medical attention?** Yes ☐**.** No ☐. If you feel that participation in this study caused any of the above or are concerned, please contact the project manager on 0434934198. If it is an emergency please call 000. |
|  | Week 5  **Date:** |
| Healthcare | 1. **Write in each column, in the corresponding day of the week, the clinician code (see page 1) you visited on this week and the kilometres travelled**  \|  \| Monday \| Tuesday \| Wednesday \| Thursday \| Friday \| Saturday \| Sunday \| \| --- \| --- \| --- \| --- \| --- \| --- \| --- \| --- \| \| **Clinician code** \|  \|  \|  \|  \|  \|  \|  \| \| **Kilometres travelled** \|  \|  \|  \|  \|  \|  \|  \|   For clinician code please see **page 1** |
|  |  |
| Exercises | 1. **Did you perform any exercises prescribed by a clinician on this week? (Tick all days that apply)**  \| Monday \| Tuesday \| Wednesday \| Thursday \| Friday \| Saturday \| Sunday \| \| --- \| --- \| --- \| --- \| --- \| --- \| --- \| \|  \|  \|  \|  \|  \|  \|  \| |
|  |  |
| Physical activity | 1. **Write in each column, in the corresponding day of the week, the physical activity code (see page 1) you performed for more than 10 minutes and the time in minutes for each activity on this week:**  \|  \| Monday \| Tuesday \| Wednesday \| Thursday \| Friday \| Saturday \| Sunday \| \| --- \| --- \| --- \| --- \| --- \| --- \| --- \| --- \| \| **Activity Code** \|  \|  \|  \|  \|  \|  \|  \| \| **Minutes** \|  \|  \|  \|  \|  \|  \|  \|   For activity codes please see **page 1** |
| F |  |
| Medication | 1. **Write in each column a medication name, strength per unit (e.g. 500 mg) please specify milligrams (mg) or grams (g) and the number of pills taken on this week:**  \|  \| Monday \| Tuesday \| Wednesday \| Thursday \| Friday \| Saturday \| Sunday \| \| --- \| --- \| --- \| --- \| --- \| --- \| --- \| --- \| \| **Medication name** \|  \|  \|  \|  \|  \|  \|  \| \| **Strength p.u.** \|  \|  \|  \|  \|  \|  \|  \| \| **N. of pills per day** \|  \|  \|  \|  \|  \|  \|  \| |
|  |  |
| Adverse events | Please add a tick (✔) to as many as appropriate:  **Did you experience any of the following this week?**  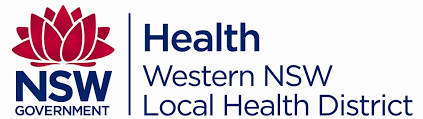Increased back pain ☐. Increased knee pain ☐. Pain elsewhere ☐. Muscle soreness ☐. Swelling ☐. Cramp ☐.  Trip/fall ☐. Serious event ☐. Other symptoms ☐  **Did any of the above last more than 24 hours?** Yes ☐**.** No ☐.  **Did you require medical attention?** Yes ☐**.** No ☐. If you feel that participation in this study caused any of the above or are concerned, please contact the project manager on 0434934198. If it is an emergency please call 000. |
|  | Week 6  **Date:** |
| Healthcare | 1. **Write in each column, in the corresponding day of the week, the clinician code (see page 1) you visited on this week and the kilometres travelled**  \|  \| Monday \| Tuesday \| Wednesday \| Thursday \| Friday \| Saturday \| Sunday \| \| --- \| --- \| --- \| --- \| --- \| --- \| --- \| --- \| \| **Clinician code** \|  \|  \|  \|  \|  \|  \|  \| \| **Kilometres travelled** \|  \|  \|  \|  \|  \|  \|  \|   For clinician code please see **page 1** |
|  |  |
| Exercises | 1. **Did you perform any exercises prescribed by a clinician on this week? (Tick all days that apply)**  \| Monday \| Tuesday \| Wednesday \| Thursday \| Friday \| Saturday \| Sunday \| \| --- \| --- \| --- \| --- \| --- \| --- \| --- \| \|  \|  \|  \|  \|  \|  \|  \| |
|  |  |
| Physical activity | 1. **Write in each column, in the corresponding day of the week, the physical activity code (see page 1) you performed for more than 10 minutes and the time in minutes for each activity on this week:**  \|  \| Monday \| Tuesday \| Wednesday \| Thursday \| Friday \| Saturday \| Sunday \| \| --- \| --- \| --- \| --- \| --- \| --- \| --- \| --- \| \| **Activity Code** \|  \|  \|  \|  \|  \|  \|  \| \| **Minutes** \|  \|  \|  \|  \|  \|  \|  \|   For activity codes please see **page 1** |
| F |  |
| Medication | 1. **Write in each column a medication name, strength per unit (e.g. 500 mg) please specify milligrams (mg) or grams (g) and the number of pills taken on this week:**  \|  \| Monday \| Tuesday \| Wednesday \| Thursday \| Friday \| Saturday \| Sunday \| \| --- \| --- \| --- \| --- \| --- \| --- \| --- \| --- \| \| **Medication name** \|  \|  \|  \|  \|  \|  \|  \| \| **Strength p.u.** \|  \|  \|  \|  \|  \|  \|  \| \| **N. of pills per day** \|  \|  \|  \|  \|  \|  \|  \| |
|  |  |
| Adverse events | Please add a tick (✔) to as many as appropriate:  **Did you experience any of the following this week?**  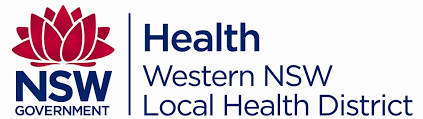Increased back pain ☐. Increased knee pain ☐. Pain elsewhere ☐. Muscle soreness ☐. Swelling ☐. Cramp ☐.  Trip/fall ☐. Serious event ☐. Other symptoms ☐  **Did any of the above last more than 24 hours?** Yes ☐**.** No ☐.  **Did you require medical attention?** Yes ☐**.** No ☐. If you feel that participation in this study caused any of the above or are concerned, please contact the project manager on 0434934198. If it is an emergency please call 000. |
|  | Week 7  **Date:** |
| Healthcare | 1. **Write in each column, in the corresponding day of the week, the clinician code (see page 1) you visited on this week and the kilometres travelled**  \|  \| Monday \| Tuesday \| Wednesday \| Thursday \| Friday \| Saturday \| Sunday \| \| --- \| --- \| --- \| --- \| --- \| --- \| --- \| --- \| \| **Clinician code** \|  \|  \|  \|  \|  \|  \|  \| \| **Kilometres travelled** \|  \|  \|  \|  \|  \|  \|  \|   For clinician code please see **page 1** |
|  |  |
| Exercises | 1. **Did you perform any exercises prescribed by a clinician on this week? (Tick all days that apply)**  \| Monday \| Tuesday \| Wednesday \| Thursday \| Friday \| Saturday \| Sunday \| \| --- \| --- \| --- \| --- \| --- \| --- \| --- \| \|  \|  \|  \|  \|  \|  \|  \| |
|  |  |
| Physical activity | 1. **Write in each column, in the corresponding day of the week, the physical activity code (see page 1) you performed for more than 10 minutes and the time in minutes for each activity on this week:**  \|  \| Monday \| Tuesday \| Wednesday \| Thursday \| Friday \| Saturday \| Sunday \| \| --- \| --- \| --- \| --- \| --- \| --- \| --- \| --- \| \| **Activity Code** \|  \|  \|  \|  \|  \|  \|  \| \| **Minutes** \|  \|  \|  \|  \|  \|  \|  \|   For activity codes please see **page 1** |
| F |  |
| Medication | 1. **Write in each column a medication name, strength per unit (e.g. 500 mg) please specify milligrams (mg) or grams (g) and the number of pills taken on this week:**  \|  \| Monday \| Tuesday \| Wednesday \| Thursday \| Friday \| Saturday \| Sunday \| \| --- \| --- \| --- \| --- \| --- \| --- \| --- \| --- \| \| **Medication name** \|  \|  \|  \|  \|  \|  \|  \| \| **Strength p.u.** \|  \|  \|  \|  \|  \|  \|  \| \| **N. of pills per day** \|  \|  \|  \|  \|  \|  \|  \| |
|  |  |
| Adverse events | Please add a tick (✔) to as many as appropriate:  **Did you experience any of the following this week?**  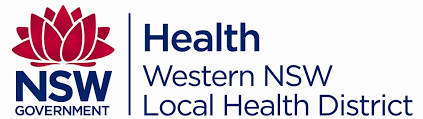Increased back pain ☐. Increased knee pain ☐. Pain elsewhere ☐. Muscle soreness ☐. Swelling ☐. Cramp ☐.  Trip/fall ☐. Serious event ☐. Other symptoms ☐  **Did any of the above last more than 24 hours?** Yes ☐**.** No ☐.  **Did you require medical attention?** Yes ☐**.** No ☐. If you feel that participation in this study caused any of the above or are concerned, please contact the project manager on 0434934198. If it is an emergency please call 000. |
|  | Week 8  **Date:** |
| Healthcare | 1. **Write in each column, in the corresponding day of the week, the clinician code (see page 1) you visited on this week and the kilometres travelled**  \|  \| Monday \| Tuesday \| Wednesday \| Thursday \| Friday \| Saturday \| Sunday \| \| --- \| --- \| --- \| --- \| --- \| --- \| --- \| --- \| \| **Clinician code** \|  \|  \|  \|  \|  \|  \|  \| \| **Kilometres travelled** \|  \|  \|  \|  \|  \|  \|  \|   For clinician code please see **page 1** |
|  |  |
| Exercises | 1. **Did you perform any exercises prescribed by a clinician on this week? (Tick all days that apply)**  \| Monday \| Tuesday \| Wednesday \| Thursday \| Friday \| Saturday \| Sunday \| \| --- \| --- \| --- \| --- \| --- \| --- \| --- \| \|  \|  \|  \|  \|  \|  \|  \| |
|  |  |
| Physical activity | 1. **Write in each column, in the corresponding day of the week, the physical activity code (see page 1) you performed for more than 10 minutes and the time in minutes for each activity on this week:**  \|  \| Monday \| Tuesday \| Wednesday \| Thursday \| Friday \| Saturday \| Sunday \| \| --- \| --- \| --- \| --- \| --- \| --- \| --- \| --- \| \| **Activity Code** \|  \|  \|  \|  \|  \|  \|  \| \| **Minutes** \|  \|  \|  \|  \|  \|  \|  \|   For activity codes please see **page 1** |
| F |  |
| Medication | 1. **Write in each column a medication name, strength per unit (e.g. 500 mg) please specify milligrams (mg) or grams (g) and the number of pills taken on this week:**  \|  \| Monday \| Tuesday \| Wednesday \| Thursday \| Friday \| Saturday \| Sunday \| \| --- \| --- \| --- \| --- \| --- \| --- \| --- \| --- \| \| **Medication name** \|  \|  \|  \|  \|  \|  \|  \| \| **Strength p.u.** \|  \|  \|  \|  \|  \|  \|  \| \| **N. of pills per day** \|  \|  \|  \|  \|  \|  \|  \| |
|  |  |
| Adverse events | Please add a tick (✔) to as many as appropriate:  **Did you experience any of the following this week?**  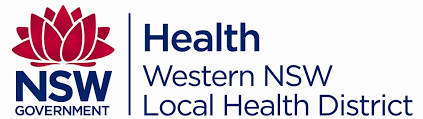Increased back pain ☐. Increased knee pain ☐. Pain elsewhere ☐. Muscle soreness ☐. Swelling ☐. Cramp ☐.  Trip/fall ☐. Serious event ☐. Other symptoms ☐  **Did any of the above last more than 24 hours?** Yes ☐**.** No ☐.  **Did you require medical attention?** Yes ☐**.** No ☐. If you feel that participation in this study caused any of the above or are concerned, please contact the project manager on 0434934198. If it is an emergency please call 000. |
|  | Week 9  **Date:** |
| Healthcare | 1. **Write in each column, in the corresponding day of the week, the clinician code (see page 1) you visited on this week and the kilometres travelled**  \|  \| Monday \| Tuesday \| Wednesday \| Thursday \| Friday \| Saturday \| Sunday \| \| --- \| --- \| --- \| --- \| --- \| --- \| --- \| --- \| \| **Clinician code** \|  \|  \|  \|  \|  \|  \|  \| \| **Kilometres travelled** \|  \|  \|  \|  \|  \|  \|  \|   For clinician code please see **page 1** |
|  |  |
| Exercises | 1. **Did you perform any exercises prescribed by a clinician on this week? (Tick all days that apply)**  \| Monday \| Tuesday \| Wednesday \| Thursday \| Friday \| Saturday \| Sunday \| \| --- \| --- \| --- \| --- \| --- \| --- \| --- \| \|  \|  \|  \|  \|  \|  \|  \| |
|  |  |
| Physical activity | 1. **Write in each column, in the corresponding day of the week, the physical activity code (see page 1) you performed for more than 10 minutes and the time in minutes for each activity on this week:**  \|  \| Monday \| Tuesday \| Wednesday \| Thursday \| Friday \| Saturday \| Sunday \| \| --- \| --- \| --- \| --- \| --- \| --- \| --- \| --- \| \| **Activity Code** \|  \|  \|  \|  \|  \|  \|  \| \| **Minutes** \|  \|  \|  \|  \|  \|  \|  \|   For activity codes please see **page 1** |
| F |  |
| Medication | 1. **Write in each column a medication name, strength per unit (e.g. 500 mg) please specify milligrams (mg) or grams (g) and the number of pills taken on this week:**  \|  \| Monday \| Tuesday \| Wednesday \| Thursday \| Friday \| Saturday \| Sunday \| \| --- \| --- \| --- \| --- \| --- \| --- \| --- \| --- \| \| **Medication name** \|  \|  \|  \|  \|  \|  \|  \| \| **Strength p.u.** \|  \|  \|  \|  \|  \|  \|  \| \| **N. of pills per day** \|  \|  \|  \|  \|  \|  \|  \| |
|  |  |
| Adverse events | Please add a tick (✔) to as many as appropriate:  **Did you experience any of the following this week?**  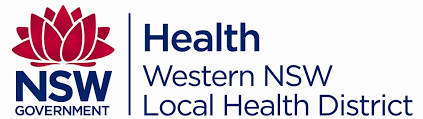Increased back pain ☐. Increased knee pain ☐. Pain elsewhere ☐. Muscle soreness ☐. Swelling ☐. Cramp ☐.  Trip/fall ☐. Serious event ☐. Other symptoms ☐  **Did any of the above last more than 24 hours?** Yes ☐**.** No ☐.  **Did you require medical attention?** Yes ☐**.** No ☐. If you feel that participation in this study caused any of the above or are concerned, please contact the project manager on 0434934198. If it is an emergency please call 000. |
|  | Week 10  **Date:** |
| Healthcare | 1. **Write in each column, in the corresponding day of the week, the clinician code (see page 1) you visited on this week and the kilometres travelled**  \|  \| Monday \| Tuesday \| Wednesday \| Thursday \| Friday \| Saturday \| Sunday \| \| --- \| --- \| --- \| --- \| --- \| --- \| --- \| --- \| \| **Clinician code** \|  \|  \|  \|  \|  \|  \|  \| \| **Kilometres travelled** \|  \|  \|  \|  \|  \|  \|  \|   For clinician code please see **page 1** |
|  |  |
| Exercises | 1. **Did you perform any exercises prescribed by a clinician on this week? (Tick all days that apply)**  \| Monday \| Tuesday \| Wednesday \| Thursday \| Friday \| Saturday \| Sunday \| \| --- \| --- \| --- \| --- \| --- \| --- \| --- \| \|  \|  \|  \|  \|  \|  \|  \| |
|  |  |
| Physical activity | 1. **Write in each column, in the corresponding day of the week, the physical activity code (see page 1) you performed for more than 10 minutes and the time in minutes for each activity on this week:**  \|  \| Monday \| Tuesday \| Wednesday \| Thursday \| Friday \| Saturday \| Sunday \| \| --- \| --- \| --- \| --- \| --- \| --- \| --- \| --- \| \| **Activity Code** \|  \|  \|  \|  \|  \|  \|  \| \| **Minutes** \|  \|  \|  \|  \|  \|  \|  \|   For activity codes please see **page 1** |
| F |  |
| Medication | 1. **Write in each column a medication name, strength per unit (e.g. 500 mg) please specify milligrams (mg) or grams (g) and the number of pills taken on this week:**  \|  \| Monday \| Tuesday \| Wednesday \| Thursday \| Friday \| Saturday \| Sunday \| \| --- \| --- \| --- \| --- \| --- \| --- \| --- \| --- \| \| **Medication name** \|  \|  \|  \|  \|  \|  \|  \| \| **Strength p.u.** \|  \|  \|  \|  \|  \|  \|  \| \| **N. of pills per day** \|  \|  \|  \|  \|  \|  \|  \| |
|  |  |
| Adverse events | Please add a tick (✔) to as many as appropriate:  **Did you experience any of the following this week?**  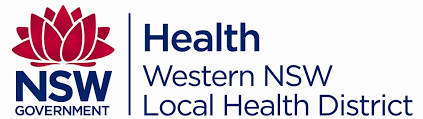Increased back pain ☐. Increased knee pain ☐. Pain elsewhere ☐. Muscle soreness ☐. Swelling ☐. Cramp ☐.  Trip/fall ☐. Serious event ☐. Other symptoms ☐  **Did any of the above last more than 24 hours?** Yes ☐**.** No ☐.  **Did you require medical attention?** Yes ☐**.** No ☐. If you feel that participation in this study caused any of the above or are concerned, please contact the project manager on 0434934198. If it is an emergency please call 000. |
|  | Week 11  **Date:** |
| Healthcare | 1. **Write in each column, in the corresponding day of the week, the clinician code (see page 1) you visited on this week and the kilometres travelled**  \|  \| Monday \| Tuesday \| Wednesday \| Thursday \| Friday \| Saturday \| Sunday \| \| --- \| --- \| --- \| --- \| --- \| --- \| --- \| --- \| \| **Clinician code** \|  \|  \|  \|  \|  \|  \|  \| \| **Kilometres travelled** \|  \|  \|  \|  \|  \|  \|  \|   For clinician code please see **page 1** |
|  |  |
| Exercises | 1. **Did you perform any exercises prescribed by a clinician on this week? (Tick all days that apply)**  \| Monday \| Tuesday \| Wednesday \| Thursday \| Friday \| Saturday \| Sunday \| \| --- \| --- \| --- \| --- \| --- \| --- \| --- \| \|  \|  \|  \|  \|  \|  \|  \| |
|  |  |
| Physical activity | 1. **Write in each column, in the corresponding day of the week, the physical activity code (see page 1) you performed for more than 10 minutes and the time in minutes for each activity on this week:**  \|  \| Monday \| Tuesday \| Wednesday \| Thursday \| Friday \| Saturday \| Sunday \| \| --- \| --- \| --- \| --- \| --- \| --- \| --- \| --- \| \| **Activity Code** \|  \|  \|  \|  \|  \|  \|  \| \| **Minutes** \|  \|  \|  \|  \|  \|  \|  \|   For activity codes please see **page 1** |
| F |  |
| Medication | 1. **Write in each column a medication name, strength per unit (e.g. 500 mg) please specify milligrams (mg) or grams (g) and the number of pills taken on this week:**  \|  \| Monday \| Tuesday \| Wednesday \| Thursday \| Friday \| Saturday \| Sunday \| \| --- \| --- \| --- \| --- \| --- \| --- \| --- \| --- \| \| **Medication name** \|  \|  \|  \|  \|  \|  \|  \| \| **Strength p.u.** \|  \|  \|  \|  \|  \|  \|  \| \| **N. of pills per day** \|  \|  \|  \|  \|  \|  \|  \| |
|  |  |
| Adverse events | Please add a tick (✔) to as many as appropriate:  **Did you experience any of the following this week?**  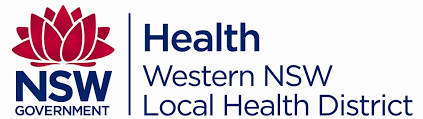Increased back pain ☐. Increased knee pain ☐. Pain elsewhere ☐. Muscle soreness ☐. Swelling ☐. Cramp ☐.  Trip/fall ☐. Serious event ☐. Other symptoms ☐  **Did any of the above last more than 24 hours?** Yes ☐**.** No ☐.  **Did you require medical attention?** Yes ☐**.** No ☐. If you feel that participation in this study caused any of the above or are concerned, please contact the project manager on 0434934198. If it is an emergency please call 000. |
|  | Week 12  **Date:** |
| Healthcare | 1. **Write in each column, in the corresponding day of the week, the clinician code (see page 1) you visited on this week and the kilometres travelled**  \|  \| Monday \| Tuesday \| Wednesday \| Thursday \| Friday \| Saturday \| Sunday \| \| --- \| --- \| --- \| --- \| --- \| --- \| --- \| --- \| \| **Clinician code** \|  \|  \|  \|  \|  \|  \|  \| \| **Kilometres travelled** \|  \|  \|  \|  \|  \|  \|  \|   For clinician code please see **page 1** |
|  |  |
| Exercises | 1. **Did you perform any exercises prescribed by a clinician on this week? (Tick all days that apply)**  \| Monday \| Tuesday \| Wednesday \| Thursday \| Friday \| Saturday \| Sunday \| \| --- \| --- \| --- \| --- \| --- \| --- \| --- \| \|  \|  \|  \|  \|  \|  \|  \| |
|  |  |
| Physical activity | 1. **Write in each column, in the corresponding day of the week, the physical activity code (see page 1) you performed for more than 10 minutes and the time in minutes for each activity on this week:**  \|  \| Monday \| Tuesday \| Wednesday \| Thursday \| Friday \| Saturday \| Sunday \| \| --- \| --- \| --- \| --- \| --- \| --- \| --- \| --- \| \| **Activity Code** \|  \|  \|  \|  \|  \|  \|  \| \| **Minutes** \|  \|  \|  \|  \|  \|  \|  \|   For activity codes please see **page 1** |
| F |  |
| Medication | 1. **Write in each column a medication name, strength per unit (e.g. 500 mg) please specify milligrams (mg) or grams (g) and the number of pills taken on this week:**  \|  \| Monday \| Tuesday \| Wednesday \| Thursday \| Friday \| Saturday \| Sunday \| \| --- \| --- \| --- \| --- \| --- \| --- \| --- \| --- \| \| **Medication name** \|  \|  \|  \|  \|  \|  \|  \| \| **Strength p.u.** \|  \|  \|  \|  \|  \|  \|  \| \| **N. of pills per day** \|  \|  \|  \|  \|  \|  \|  \| |
|  |  |
| Adverse events | Please add a tick (✔) to as many as appropriate:  **Did you experience any of the following this week?**  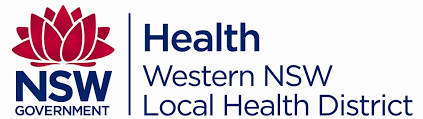Increased back pain ☐. Increased knee pain ☐. Pain elsewhere ☐. Muscle soreness ☐. Swelling ☐. Cramp ☐.  Trip/fall ☐. Serious event ☐. Other symptoms ☐  **Did any of the above last more than 24 hours?** Yes ☐**.** No ☐.  **Did you require medical attention?** Yes ☐**.** No ☐. If you feel that participation in this study caused any of the above or are concerned, please contact the project manager on 0434934198. If it is an emergency please call 000. |

**How to send a picture of the logbook on an email?**

1. Open the camera application on your phone or tablet and take a clear picture of the entire page once you fill it up the whole week.
2. Go to the main menu and open your mail account (Gmail, Lives, outlook, etc)
3. Click on compose an email
4. ‘To’: [empower](mailto:Antonio.michell@sydney.edu.au).study@sydney.edu.au
5. ‘Subject’: please write your name and the page number of the logbook you are sending us.
6. Android: Tap on the button attach a file (paper clip symbol)
7. Iphone: double-tap the text field of the email message.
8. The stripe with extra options appears. Tap the little triangle on the right of the stripe to invoke extra options. Tap "Insert Photo or Video". The" Mail" app will redirect you to your Photos. Tap the album you're interested in and choose the picture you want to send.
9. Choose the picture of the logbook you just took
10. You also can attach several pictures pressing for a couple of seconds on the image and selecting the next.
11. Tap "Choose" to confirm the choice of the photo. Back to our unfinished message - tap "Send".

| **Android** | **Iphone** |
| --- | --- |
| 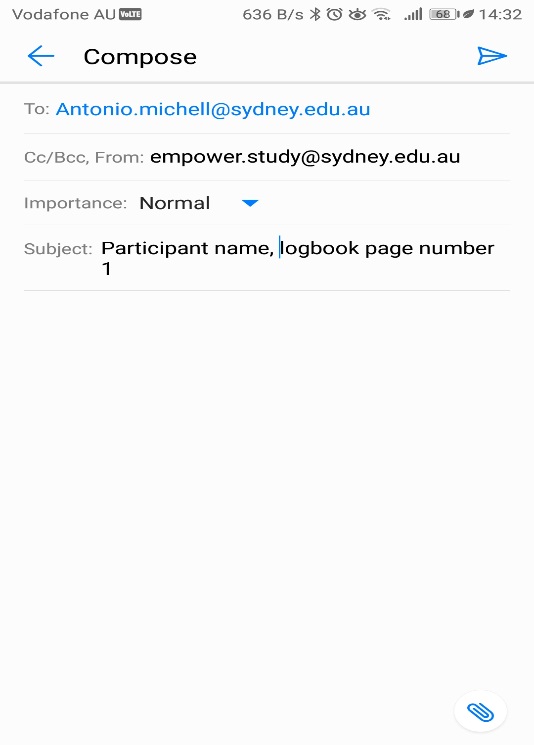 [empower](mailto:Antonio.michell@sydney.edu.au).study@sydney.edu.au | 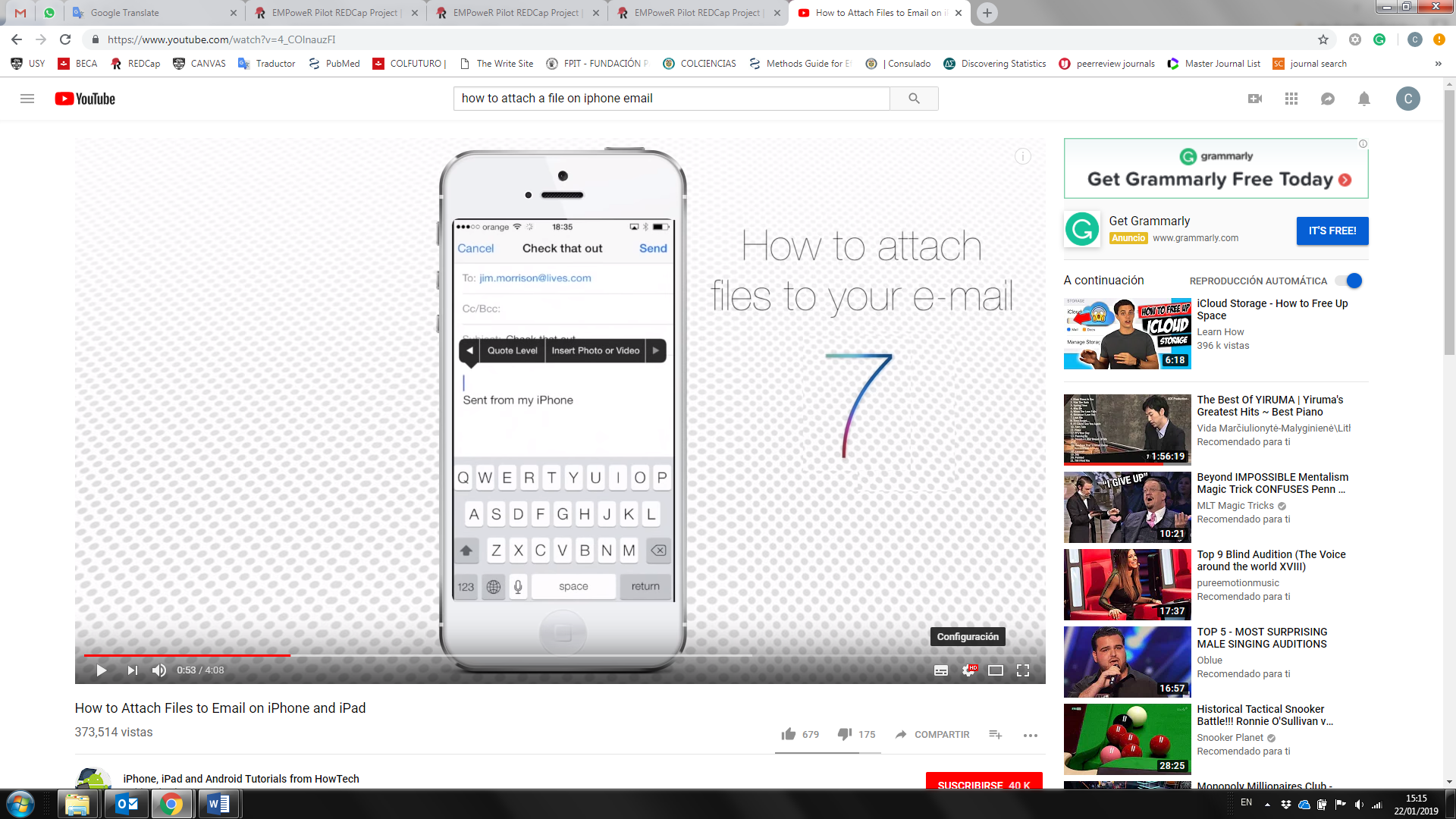 [empower](mailto:Antonio.michell@sydney.edu.au).study@sydney.edu.au |
